# Chemically modified MIR143-3p exhibited anti-cancer effects by impairing the KRAS network in colorectal cancer cells

**DOI:** 10.1016/j.omtn.2022.09.001

**Published:** 2022-09-07

**Authors:** Nobuhiko Sugito, Kazuki Heishima, Yukihiro Akao

**Affiliations:** 1 United Graduate School of Drug Discovery and Medical Information Sciences, Gifu University, 1-1 Yanagido, Gifu 501-1194, Japan; 2 Gifu University Institute for Advanced Study, 1-1 Yanagido, Gifu 501-1193, Japan

**Keywords:** MT: non-coding RNAs, KRAS mutation, colorectal cancer, cancer, microRNA, miR-143-3p

## Abstract

*Kirsten rat sarcoma virus* (*KRAS*) mutations are frequently detected in many cancers and are major driver genes. Therefore, KRAS inhibitors have been the subject of extensive research. We developed chemically modified MIR143-3p (MIR143#12), which exhibits higher anticancer activity and nuclease resistance than other commercial inhibitors. MIR143#12 potently suppressed cell growth in colorectal and pancreatic cancer cell lines via apoptosis induced by repression of the entire rat sarcoma virus (RAS) network, which was achieved by silencing *KRAS*, *Son of sevenless homolog 1 (SOS1)*, *AKT*, and extracellular signal-regulated kinase (*ERK*). We investigated the mechanistic advantages of MIR143#12 in various KRAS mutant colorectal cancer cell lines. Its effects were stronger than those of knockdown of *KRAS* alone in colon cancer cells because silencing of *KRAS* by small interfering RNA (siRNA) did not decrease the protein expression levels of AKT or ERKs. The *KRAS* mRNA recruitment system, called the “positive circuit” under effector signaling pathways, may contribute to insensitivity of KRAS mutant cancers to MIR143#12 and siRNAs. In an *in vivo* study, we newly demonstrated that MIR143#12 induced neoangiogenesis in the tumor microenvironment with growth suppression. Based on the present results, it is crucial to down-regulate not only KRAS but also the entire KRAS signaling network, which may be accomplished by MIR143#12.

## Graphical abstract

**Figure:**
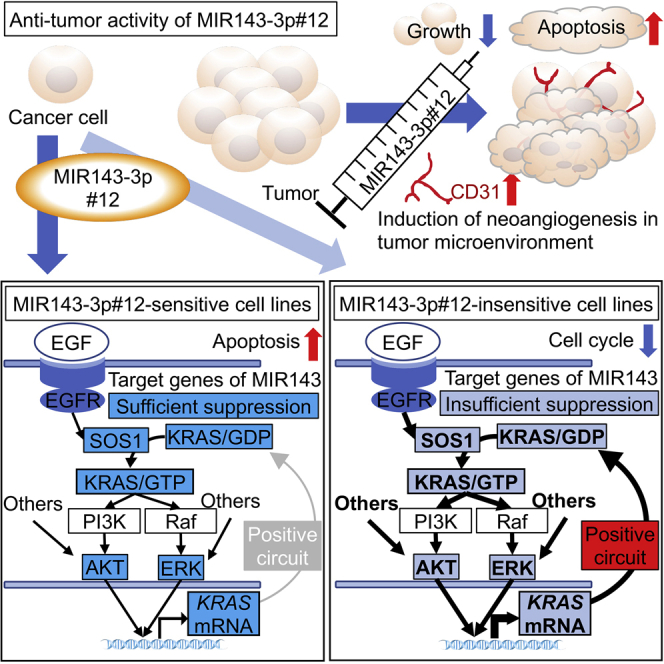

## Teaser

Suppression of the entire KRAS network by MIR143#12 showed stronger anticancer activity in KRAS mutant colorectal cancer cell lines than suppression of individual genes in the KRAS network, such as KRAS, AKT, and ERK. MIR143#12 replacement therapy is a very effective treatment strategy for KRAS mutant colorectal cancer.

## Introduction

Rat sarcoma virus (RAS) mutations are genetic events that have been detected in approximately 30% of all human cancers, with the specific RAS isoform generally differing according to the cancer type.^1^ Mutations in *Kirsten rat sarcoma virus* (*KRAS*) account for approximately 85% of all RAS mutations.^2^ Oncogenic mutations in *KRAS* genes have been reported in approximately 40% of human colon malignancies and 90% of all pancreatic cancer cases.^3^ The epidermal growth factor receptor (EGFR) is an important molecule involved in cancer biology and therapy.^4^ KRAS, an essential component of the EGFR signaling cascade, activates the downstream growth-related signaling pathways rapidly accelerated fibrosarcoma (Raf)-mitogen-activated protein kinase (MAPK) and phosphatidylinositol 3-kinase (PI3K)/AKT.^5^ Cetuximab, an immunoglobulin G1 (IgG1) chimeric monoclonal antibody against EGFR, has been shown to prolong overall survival and progression-free survival in individuals with metastatic colorectal cancer who did not respond to chemotherapy. However, the efficacy of cetuximab is currently limited to individuals with wild-type KRAS tumors because mutant KRAS tumors do not respond to cetuximab.^6^ Various efforts have been made to develop one or more small-molecule compounds that directly target and constitutively inhibit active KRAS; however, the lack of druggable pockets on the surface of RAS has made their development difficult.^7^ An inhibitor that targets KRAS (G12C) has been approved for treatment of non-small cell lung cancer (NSCLC).^8^ However, inhibitors for other mutations have not yet been developed. Therefore, new strategies and technologies for KRAS mutant cancers are needed.

In recent years, microRNA (miRNA, MIR), a small nucleic acid molecule, has begun to revolutionize the world of drug discovery.^9^ Decreases in the intracellular levels of certain miRNAs have been shown to induce transmission of genetic information, leading to development of diseases.^10^ We have reported previously that expression of the tumor suppressor MIR143, which is mainly transcribed by p53,^11^^,^^12^ is lower in approximately 80% of human colorectal tumor samples from individuals with cancer and adenoma than in normal tissues.^13^ Expression of MIR143 is lower in pancreatic ductal adenocarcinoma tissues than in paired adjacent normal tissues.^14^ The basic concept of miRNA therapy is restoration of normal conditions through replacement of down-regulated miRNA, tumor suppressor miRNA, in tumor cells. We recently demonstrated that chemically modified (CM) MIR143#12 significantly inhibited cancer cell growth by targeting *KRAS*, *Son of sevenless homolog 1* (*SOS1*), *AKT*, and *extracellular signal-regulated kinase* (*ERK*) in colorectal cancer,^15^ bladder cancer,^16^ gastric cancer,^17^ and rhabdomyosarcoma cells.^18^ Using MIR143#12, which has 2 binding sites in the 3′ UTR of *KRAS*, we clearly identified target genes related to the KRAS network and the KRAS “positive circuit,” the recruitment system for *KRAS* mRNA from the PI3K/AKT and MAPK signaling pathways, in colorectal tumor cells.^15^

In the present study, we investigated the mechanistic advantages of MIR143#12 against KRAS mutant cancer cells. The results showed that MIR143#12 exerted anticancer effects on colorectal and pancreatic cancer cells with or without KRAS mutations by inhibiting the entire KRAS network. These effects were stronger than those of knockdown of *KRAS*, *AKT*, or *ERK* alone. Therefore, MIR143#12 has potential as a therapeutic agent for cancers in which KRAS or KRAS-related genes are mutated.

## Results

### CM MIR143-3p#12 exhibited higher anticancer activity and nuclease resistance

**Figure 1:**
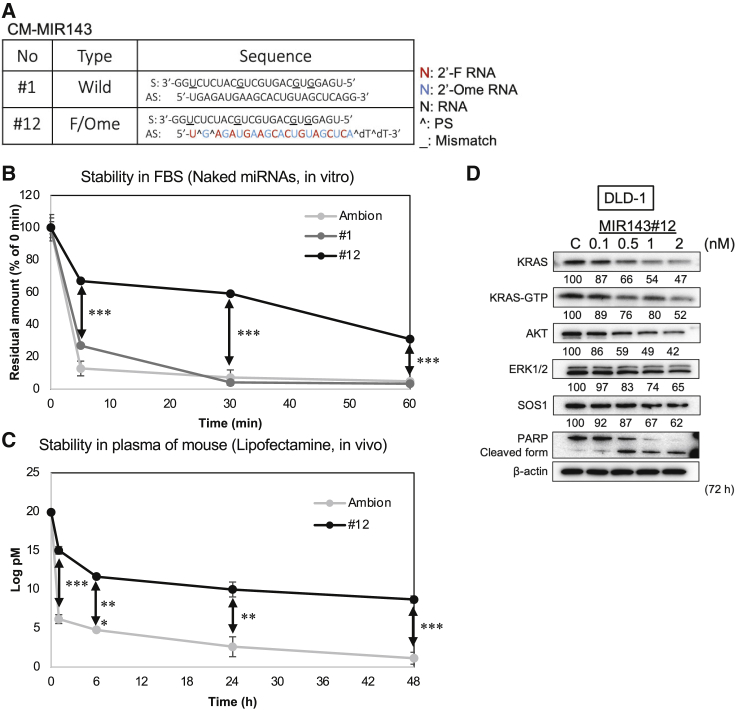
Chemically modified (CM) MIR143-3p#12 has high nuclease resistance and anticancer activity (A) RNA sequences of CM MIR143#1 is the wild type of MIR143. MIR143#12 is F/Ome-modified MIR143. Three mismatched nucleic acids of the sense strand are underlined. F RNA, Fluoro-RNA; Ome RNA, O-methyl RNA; PS, phosphorothioate. (B) The remaining percentages of each naked MIR143, Ambion (Applied Biosystems, Foster City, CA, USA), MIR143#1, or MIR143#12 remaining in the presence of FBS were evaluated by qRT-PCR. The 0-min value of each MIR143 is indicated as 100%. The mean value was taken for each time. (C) The residual amount of MIR143, Ambion, or MIR143#12 remaining in the blood of mice was evaluated by qRT-PCR. MIR143 was mixed with Lipofectamine RNAiMAX reagent and injected intravenously into mice. Blood samples were collected after 1, 6, 24, and 48 h. The mean value was taken for each time. (D) The RNA interference of MIR143#12 was evaluated by expression of target genes in DLD-1 cell lines. The apoptosis marker cleaved form of PARP was confirmed by western blot analysis. Results are shown as the mean ± SD; ∗∗p < 0.01, ∗∗∗p < 0.001.

To examine the potential of MIR143 as a KRAS inhibitor against KRAS mutant cancer cells, we designed and synthesized more than 120 CM MIR143s with double-stranded structures with high nuclease resistance and anticancer activity.^15^ We focused on a MIR143 derivative. The structure of the derivative, MIR143#12, is shown in Figure 1A. MIR143#12 was chosen because it had the greatest growth-inhibitory activity and RNase resistance among more than 120 CM MIR143s. MIR143#1 has the same double-stranded RNA sequence as that found in wild-type mature MIR143. In contrast to MIR143#1, the antisense strand of MIR143#12 was CM. We initially investigated and compared the RNase resistance of MIR143#1 and MIR143#12 with that of Ambion’s commercially available MIR143 (Am143), which was used as the standard MIR143 mimic in the present study. As shown in Figure 1B, naked MIR143#12 was significantly more stable than Am143 and MIR143#1, remaining at nearly 40% after incubation for 60 min in fetal bovine serum (FBS). When Am143 and MIR143#12 encapsulated in Lipofectamine were intravenously injected into mice to verify their stability in blood, the stability of MIR143#12 was 450-fold higher than that of Am143 after 1 h and continued to show higher stability even after 48 h (Figure 1C). Therefore, MIR143#12 exhibited a high level of resistance to RNA nuclease. The protein expression levels of SOS1, KRAS, AKT, and ERK1/2, the target genes of MIR143#12, decreased in a dose-dependent manner up to 2 nM 72 h after transfection. MIR143#12 also markedly decreased the levels of active KRAS-GTP at 72 h (Figure 1D).

### MIR143#12 inhibited the growth of colorectal and pancreatic cancer cells with or without KRAS mutations

**Figure 2:**
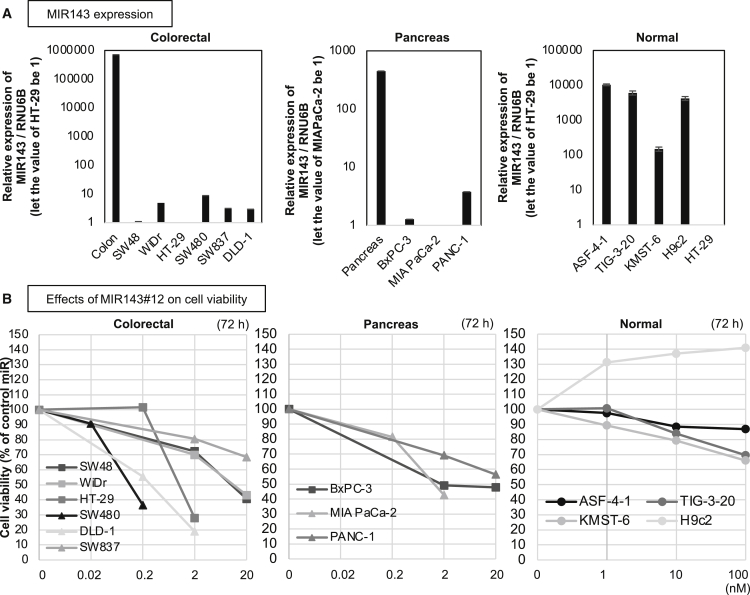
MIR143#12 replacement inhibited growth of colorectal and pancreatic cancer cells with or without KRAS mutations (A) Relative expression levels of MIR143 in normal colon or pancreas tissues and each cancer or normal cell line. Results are shown as the mean ± SD. (B) Effects of ectopic expression of MIR143#12 on the viability of colorectal, pancreatic cancer, or normal cell lines at 72 h.

We have reported previously that MIR143#12 down-regulates the protein expression of not only KRAS but also SOS1, AKT, and ERKs in the KRAS signaling network and induces apoptosis (Figure 1D).^15^ To compare the effects of MIR143#12 on cell growth with the targeting of single genes, we silenced key genes in the KRAS signaling network, such as *KRAS*, *AKT*, and *ERK*s, using their corresponding small interfering RNA (siRNA) in colorectal and pancreatic cancer cell lines harboring various types of KRAS-related mutations. Table 1 shows that MIR143#12 exhibited higher anticancer activity than silencing of *KRAS*, *AKT*, or *ERK* alone in the cell lines tested, except for half maximal inhibitory concentration (IC50) in SW837 and PANC-1 cells after 72 h. The effects of MIR143#12 on cell growth in the 4 normal karyotypic cell lines tested were negligible, even at concentrations higher than 100 nM (Table 1). MIR143 expression levels have been shown previously to be down-regulated in the majority of cancers.^19^ In the present study, MIR143 expression levels estimated by real-time quantitative reverse transcription PCR (qRT-PCR) were markedly lower in the colorectal or pancreatic cancer cells tested than in normal colon epithelial tissues; however, this difference was smaller in pancreatic cancer cells than in colorectal cancer cells (Figure 2A). MIR143 expression levels were markedly higher in the 4 normal karyotypic cell lines than in the HT-29 cell line (Figure 2A). Therefore, a lower dose of MIR143#12 is able to induce more antiproliferation activity in cell lines with wild-type KRAS than those with KRAS mutations (Figure 2B; Table 1). The growth suppression activity of MIR143#12 was higher in colorectal cancer cells than in pancreatic cancer cells, which may reflect the decreases induced in expression levels of MIR143. In contrast, even though cell growth was inhibited, the SW837 and PANC-1 cell lines were less sensitive to MIR143#12 at 72 h (Figures 2B and S1A). The inhibitory effect of MIR143#12 on cell proliferation in normal cell lines was extremely small (Figure 2B; Table 1). Although each siRNA for *KRAS*, *AKT*, and *ERK* also inhibited cell growth in the colorectal cancer cell lines tested, the effectiveness of siR-KRAS at concentrations of less than 2 nM was apparent among the siRNAs tested (Figures S1B–S1E), which indicated the crucial role of KRAS in the KRAS network.

**Table 1:** IC50 values of MIR143#12, siR-KRAS, siR-AKT, and siR-ERK2 against each cell line at 72 h (96 h in MIR143#12-insensitive SW837 and PANC-1 cell lines).

Cell line (mutation)			MIR143#12		siR-KRAS	siR-AKT	siR-ERK2
Colorectal				cell status for treatment			
SW48			10.19	apoptosis	>20	>20	>20
WiDr	BRAF V600E		11.17	apoptosis	>20	>20	>20
HT-29	BRAF V600E, T119S		1.00	apoptosis	>20	>20	7.35
SW480	KRAS G12V		0.11	apoptosis	0.65	>20	>20
DLD-1	KRAS G13D		0.28	apoptosis	1.29	>20	>20
SW837	KRAS G12C	72 h	316.72	cell cycle arrest autophagy	>20	>20	>20
		96 h	9.28	cell cycle arrest autophagy	0.32	4.32	0.26

**Pancreas**							

BxPC-3	BRAF V487A, V492A		0.36	apoptosis	>20	>20	>20
MIA PaCa-2	KRAS G12C		1.30	apoptosis	0.08	5.08	>20
PANC-1	KRAS G12D	72 h	63.25	cell cycle arrest autophagy	6.32	14.66	>20
		96 h	4.74	cell cycle arrest autophagy	0.2	0.61	0.98

**Normal**							

ASF-4-1	human fibroblast		>100		>20	>20	>20
TIG-3-20	human fibroblast		>100		>20	>20	>20
KMST-6	human fibroblast		>100		>20	>20	>20
H9C2	rat heart myoblast		>100		>20	>20	>20

### MIR143#12 was more effective than siR-KRAS

**Figure 3:**
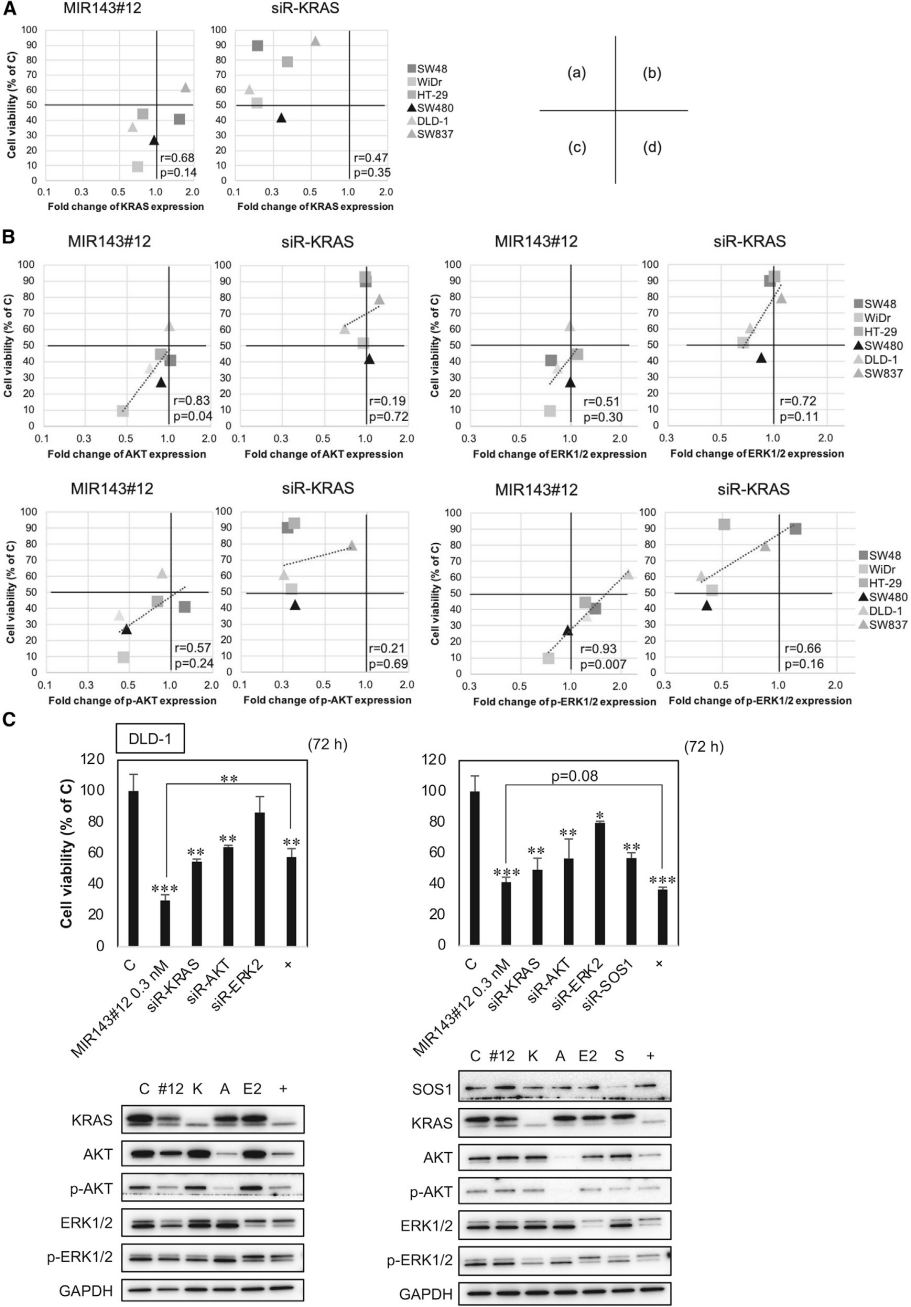
MIR143#12 was more effective than siR-KRAS in colorectal cancer cell lines (A and B) The relationship between the viable cell rate and the expression levels of KRAS (A), AKT, and ERK (B) after transfection of MIR143#12 or siR-KRAS in colorectal cancer cell lines. Concentrations of IC50 of MIR143#12 against each colorectal cancer cell line (Table 1) were used and applied to cases of siR-KRAS. Statistical analyses were performed using Pearson’s correlation coefficient and the TDIST function of Excel. p < 0.05 was considered significant. Values of r from 0.5–0.7 indicated a positive correlation and those from 0.7–1.0 a strong positive correlation. (C) A comparative experiment with MIR143#12 versus a cocktail of the above siRNAs. Results are shown as the mean ± SD; ∗p < 0.05, ∗∗p < 0.01, ∗∗∗p < 0.001.

**Figure 4:**
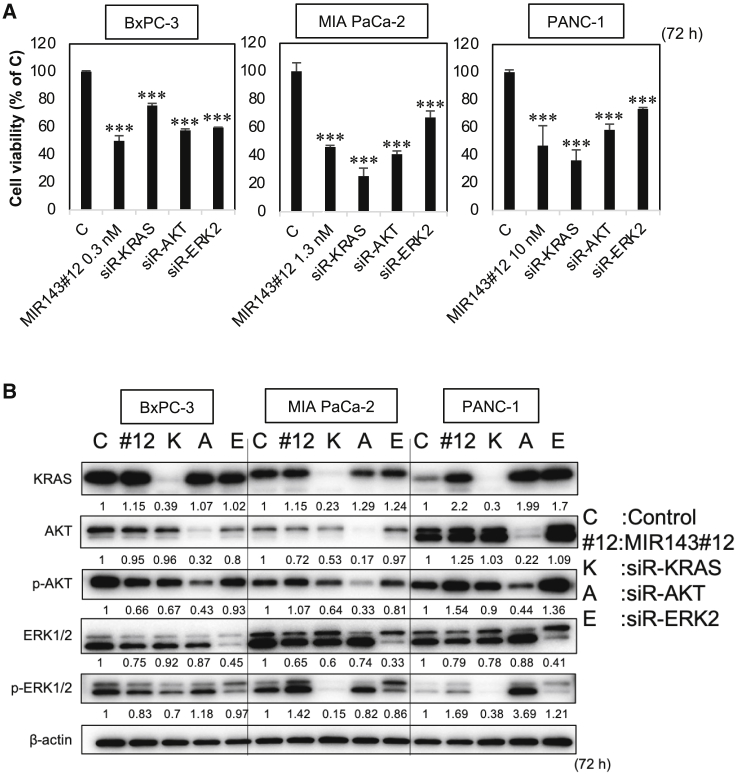
MIR143#12 and KRAS knockdown exerted similar anticancer effects in pancreatic cancer cell lines (A and B) Concentrations of IC50 of MIR143#12 against each pancreatic cancer cell line (Table 1) were used and also applied to siRNAs. (A) The effects of ectopic expression of MIR143#12, siR-KRAS, siR-AKT, and siR-ERK2 on viability were evaluated in pancreatic cancer cell lines after 72 h. Results are shown as the mean ± SD; ∗∗∗p < 0.001. (B) The effects of RNA interference by each RNA were evaluated by western blot.

We investigated the relationship between the viable cell rate and expression levels of KRAS, AKT, and ERKs after transfection of MIR143#12 or siR-KRAS in colorectal (Figures 3 and S2) and pancreatic (Figures 4 and S3) cancer cell lines. We used concentrations of IC50 of MIR143#12 against each cell line (Table 1), and the same concentration was used in each cell line to compare the anticancer activity of MIR143#12 and siRNAs. The effects of RNA interference by each siRNA were evaluated using western blotting, as shown in Figure S2B. We analyzed Figures 3 and S3 based on the cell viability in Figures 3A and S2A and the densitometry values in western blots shown in Figures 4B and S2B. Based on the results on growth inhibition by MIR143#12 or each siRNA and in view of the number of cells tested, we focused on colorectal cancer cell lines. We verified the relationship between growth suppression and the down-regulated expression of KRAS, AKT, and ERK1/2 after transfection of MIR143#12 or each siRNA. As shown in Figure 3A, a positive relationship was observed between growth suppression and the down-regulated expression of KRAS in MIR143#12-treated cells (a decrease in KRAS expression levels was associated with growth suppression; area c); however, this relationship was not detected for siR-KRAS (a decrease in the expression of KRAS did not suppress cell growth; area a). The same verification was performed for KRAS effector signaling molecules, such as AKT and ERK1/2. A positive relationship was noted between growth suppression and down-regulated expression of AKT, p-AKT, ERK1/2, and p-ERK1/2 in MIR143#12-treated cells (area c) but not for p-ERK1/2 (Figure 3B). p-ERK1/2 functioned as a survival signaling molecule because growth was inhibited by MIR143#12. A relationship was not observed between growth suppression and the down-regulated expression of siR-KRAS (Figure 3B). The down-regulated expression of p-AKT and p-ERK1/2 did not reflect suppression of growth. This analysis was not performed on pancreatic cancer cell lines because of the limited number of cells available (Figure S3). We performed a comparative experiment with MIR143#12 versus a cocktail of siRNAs. Inhibition of cell proliferation with the siRNA cocktail of *KRAS*, *AKT*, and *ERK2* was not comparable with that of MIR143#12 (Figure 3C, left panel). However, inhibition of cell proliferation using a siRNA cocktail of *KRAS*, *AKT*, *ERK2*, and *SOS1* was as effective as MIR143#12 (Figure 3C, right panel). Thus, it became apparent that the anti-cancer activity of MIR143#12 is due to suppression of the entire KRAS network. In KRAS mutant pancreatic cancer, MIR143#12 and siR-KRAS exerted similar inhibitory effects on cell proliferation (Figure 4A) and protein expression (Figure 4B). Although siR-AKT and siR-ERK2 silenced *AKT* and *ERK2*, respectively, expression of KRAS was up-regulated in the MIA PaCa-2 and PANC-1 cell lines. In PANC-1 cells, expression of p-AKT and p-ERK1/2 was up-regulated by silencing of *AKT* and *ERK2*, respectively. These results indicate that the survival system in the KRAS network functions via the positive circuit.

### The uptake of MIR143#12 into the RNA-induced silencing complex (RISC) was less efficient in MIR143#12-insensitive cell lines

**Figure 5:**
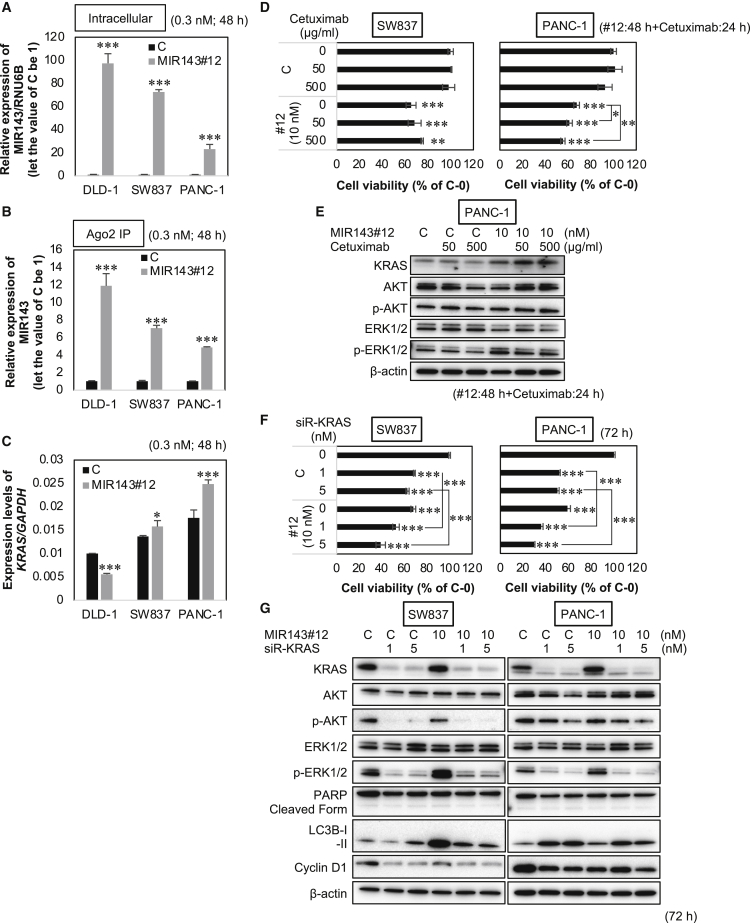
Insensitivity to MIR143#12 was overcome in MIR143#12-insensitive cancer cell lines by combining it with cetuximab or siR-KRAS (A and B) The intracellular (A) and RISC (B) uptake of MIR143#12 in the MIR143#12-sensitive cell line DLD-1 and the MIR143#12-insensitive cell lines SW837 and PANC-1 was measured using qRT-PCR after treatment with 3 nM MIR143#12 at 48 h. (C) *KRAS* mRNA expression in each of the cell lines treated with MIR143#12 was analyzed by qRT-PCR. (D and E) SW837 and PANC-1 cells were treated with control RNA or MIR143#12 for 48 hours and then treated with cetuximab for an additional 24 hours. Cell viability was estimated 72 h after transfection. Shown are the inhibitory effects of combined treatment with MIR143#12 and cetuximab on cell proliferation (D) and protein expression (E). (F and G) Inhibitory effects of combined treatment with MIR143#12 and siR-KRAS on cell proliferation (F) and protein expression (G). Results are shown as the mean ± SD; ∗p < 0.05, ∗∗p < 0.01, ∗∗∗p < 0.001.

We investigated the insensitivity of SW837 and PANC-1 cell lines to MIR143#12. Intracellular MIR143 levels were examined 48 h after transfection of MIR143#12 in MIR143#12-sensitive DLD-1 cells and MIR143#12-insensitive SW837 and PANC-1 cells. As shown in Figure 5A, the intracellular amounts of MIR143 were lower in SW837 and PANC-1 cells than in DLD-1 cells. The amounts of MIR143#12 taken into the RISC were lower in MIR143#12-insensitive SW837 and PANC-1 cells than in DLD-1 cells (Figure 5B). After transfection of MIR143#12, *KRAS* mRNA levels were significantly higher in insensitive cells than in control cells (Figure 5C). Based on these results, SW837 and PANC-1 cells appeared to be insensitive to MIR143#12 because of lower amounts of MIR143#12 being taken up into the RISC, which resulted in induction of the positive circuit.

### Insensitivity to MIR143#12 may be overcome by its combination with cetuximab or siR-KRAS

We previously reported the combined effects of MIR143#12 and the EGFR inhibitor cetuximab.^15^ Based on activation of signaling upstream of KRAS, we treated SW837 and PANC-1 cells with a combination of MIR143#12 and cetuximab. The results showed that this combination was effective in PANC-1 cells even at a low dose of cetuximab but not in SW837 cells, which have mutations in the EGFR gene even at higher doses of cetuximab (Figures 5D and 5E). Figures 4B, 5C, and S2B show that expression of KRAS was up-regulated in MIR143#12-treated SW837 and PANC-1 cells. Therefore, we treated SW837 and PANC-1 cells with a combination of MIR143#12 and siR-KRAS. As shown in Figures 5F and 5G, this combination was significantly effective. Therefore, sufficient repression of the EGFR signaling pathway to KRAS by cetuximab or down-regulation of KRAS by siRNA appears to be highly effective at overcoming insensitivity to MIR143#12. Insufficient suppression of the EGFR/KRAS network induced insensitivity to MIR143#12 in EGFR or KRAS mutant cancer cells.

### Tumor-suppressive effects of MIR143#12 in DLD-1 cell-xenografted mice

**Figure 6:**
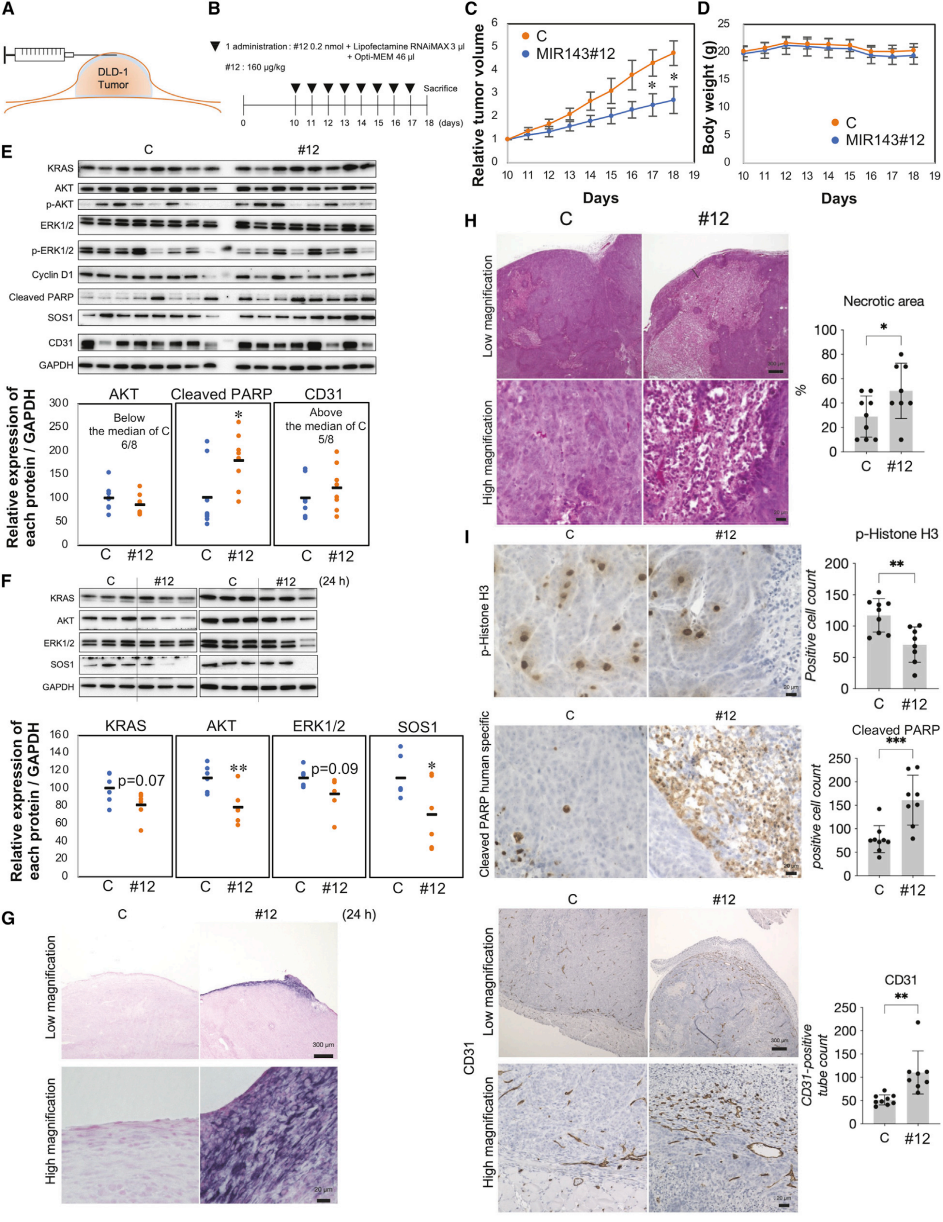
MIR143#12 exerted anti-tumor effects in nude mice (A and B) Schematic of subcutaneous administration (A) and doses and schedules (B) for DLD-1-xenografted subcutaneous tumors. (C and D) Graphs showing the time course of changes in tumor size (C) and body weight (D) in mice injected with control RNA or MIR143#12 (n = 8). (E) The top panel shows a western blot (WB) of tumor tissues treated with control RNA or MIR143#12. The bottom panel shows densitometry values obtained from WB bands used to assess expression levels. (F) The top panel shows WB detection in tumor tissues treated with one injection of control RNA or MIR143#12 for 24 h. The bottom panel shows densitometry values obtained from WB bands to assess expression levels. (G) *In situ* hybridization of MIR143 in tumor tissue treated with one injection of control RNA or MIR143#12 for 24 h. Scale bars represent 300 μm (low magnification) and 20 μm (high magnification). (H) The pathology in H&E-stained tumor tissues. Scale bars represent 300 μm (low magnification) and 20 μm (high magnification). (I) Immunostaining of tumor tissues with p-Histone H3 (top panel, scale bar represents 20 μm), cleaved PARP (center panel, scale bar represents 20 μm), and CD31 (bottom panel). Scale bars represent 300 μm (low magnification) and 20 μm (high magnification). The results of positive cells are shown as the mean ± SD; ∗p < 0.05, ∗∗p < 0.01, ∗∗∗p < 0.001.

To validate the tumor-suppressive effects of MIR143#12, we performed an *in vivo* experiment in which MIR143#12 was subcutaneously injected around the tumor for 8 consecutive days in a mouse model of subcutaneous tumor implanted with DLD-1 cells(160 μg/kg/administration; Figures 6A and 6B). To minimize the effects of the drug delivery system (DDS) on tumor growth and assess the effect of MIR143#12 on neoangiogenesis, MIR143#12 was subcutaneously injected in close proximity to tumors using lipofection. As shown in Figure 6C, the suppression of tumor growth was significantly greater in the MIR143#12-treated group than in the control group. No significant differences were observed in body weight between these groups (Figure 6D). In the western blot analysis of tumor samples, increased cleaved poly-(ADP-ribose) polymerase (PARP) was frequently detected in the MIR143#12-treated group (Figure 6E), which was consistent with the results on *in vitro* apoptotic cell death induced by MIR143#12. We also performed a western blot analysis of tumor samples collected 24 h after a single injection of MIR143#12. MIR143#12 was taken up by tumors and inhibited protein expression of target genes, such as KRAS, AKT, ERK, and SOS1 (Figure 6F). An *in situ* hybridization analysis revealed that MIR143 was taken up by tumors 24 h after a single injection of MIR143#12 (Figure 6G). Therefore, the RNA interference activity of MIR143#12 was clearly demonstrated *in vivo*. We then examined the pathology of hematoxylin and eosin (H&E)-stained tumor tissues. Low and high magnification of H&E-stained sections revealed a large number of necrotic regions in MIR143#12-treated tumors (Figure 6H). Immunostaining with p-Histone H3 and cleaved PARP showed that inhibition of cell proliferation and induction of apoptosis were stronger in MIR143#12-treated tumors than in the controls (Figure 6I). These results indicated that MIR143#12 exerted potent tumor-suppressive effects by targeting *KRAS*, *AKT*, and *ERK1/2*, even *in vivo*. Previous studies have reported that MIR143 promotes angiogenesis.^20^, ^21^, ^22^ Therefore, we validated the expression of CD31 as a marker for angiogenesis and vascular endothelial cells. A western blot analysis of tumor samples showed that expression of CD31 was up-regulated by treatment with MIR143#12 (Figure 6E). Immunostaining revealed the presence of many capillaries, which were strongly stained with the anti-CD31 antibody, particularly in the marginal regions of tumors near the site of administration of MIR143#12 (Figure 6I).

## Discussion

Here we demonstrated that CM MIR143#12, which exhibited high RNase resistance and antiproliferation activity against DLD-1 cells, induced apoptotic cell death in the colorectal and pancreatic cancer cell lines tested but did not affect the growth of normal karyotypic cell lines.

MIR143#12 exerted potent anticancer effects on colorectal cancer cells with or without KRAS mutations by inhibiting the KRAS signaling network in a complex manner that involved repression of not only *KRAS*, but also *AKT*, *ERK*, and *SOS1*. Because KRAS inhibitors still inhibit only one mutation,^23^ MIR143#12, which targets other KRAS mutations, is a promising therapeutic agent. The present study revealed that combined inhibition of the KRAS signaling network by MIR143#12 was more effective against KRAS mutant cancers than suppression of AKT, ERK, or KRAS alone.

We are currently attempting to develop miRNA medicine for KRAS mutant tumors because MIR143#12 targets multiple key genes included in the KRAS network. Expression of MIR143-3p has been shown previously to be markedly down-regulated in the majority of cancers because p53 is one of its transcription factors and functions as a tumor suppressor.^11^^,^^12^ Our strategy to overcome KRAS mutant tumors involves replacement of MIR143-3p in cancer cells. Regarding the advantages of MIR143#12, as shown in Figures 1B and 1C, naked MIR143#12 and lipoplexed MIR143#12 appeared to be stable and were still detected *in vivo* 48 h after being injected.

We demonstrated the importance of targeting multiple key genes of the KRAS network by RNA interference using MIR143#12 because the majority of KRAS mutant cancers have the KRAS recruitment system, the so-called positive circuit, as a survival system and activate it when insufficient suppressive signals enter key pathways involving KRAS, AKT, and MAPK. Other signaling pathways towards AKT or ERKs may activate this system. *KRAS* silencing by siRNA did not result in significant suppression of growth (Figure 3A); however, a positive relationship was observed between down-regulated expression and growth inhibition in MIR143#12-treated cells. The results also demonstrated that the recruitment system of KRAS may induce drug resistance. In cases where repression of the KRAS network is insufficient, growth signals into AKT or ERKs may activate the positive circuit. The two cell lines SW837 and PANC-1 were less sensitive to MIR143#12. Treatment with MIR143#12 induced cell cycle arrest and autophagic changes in both cell lines; however, the protein and mRNA expression levels of *KRAS* were up-regulated after transfection (Figure 5C). This result strongly suggests the importance of repressing the whole network without activating the positive circuit. MIR143#12 and siR-KRAS exerted synergistic effects (Figures 6C and 6D), suggesting that insufficient inhibition of the KRAS system activates the KRAS positive circuit, which contributes to insensitivity. Another reason is the low efficiency of intracellular and RISC uptake of MIR143#12 in cell lines that are insensitive to MIR143#12 (Figure 5). The sponging function of miRNAs by long non-coding RNAs (lncRNAs) has been attracting increasing attention.^24^ Previous studies have reported that several lncRNAs bound to and inhibited the function of MIR143 in a number of cancer types, including colon cancer.^25^, ^26^, ^27^, ^28^ Expression of lncRNAs sponging MIR143 was more frequent in cancer than in normal tissue.^25^, ^26^, ^27^, ^28^ Therefore, the expression of these lncRNAs may contribute to the ineffectiveness of MIR143 against cancer cells. The results shown in Table 1 indicate that chemical modifications to the antisense strand in MIR143#12 may overcome sponging by lncRNAs. Potential acquisition of resistance to KRAS (G12C) inhibitors is now recognized as an issue that needs to be resolved.^29^ Even with the most successful targeted therapies for NSCLC, such as EGFR mutant cancer, adaptive and acquired resistance pose problems for targeted therapies.^30^ This may also apply to targeted therapies that directly block molecules in the RAS-RAF-Mitogen-activated protein kinase kinase (MAP2K, MEK)-ERK pathway. This pathway is extensively regulated by a homeostatic negative feedback control that fine-tunes the pathway in cancer and normal cells. Inhibition of MEK deactivates the negative feedback mechanism and subsequently up-regulates expression of the upstream receptor tyrosine kinase (RTK).^31^ A similar process has been reported with targeted BRAF inhibition in BRAF mutant cancers, where deactivation of the negative feedback mechanism induces EGFR-mediated activation of RAS.^32^ RTK activation has also been detected after treatment with a KRAS G12C inhibitor.^33^^,^^34^ KRAS inhibitor treatment resulted in resistance because of other mutations in KRAS and activation of the bypass pathway.^29^ MIR143#12 differs from single-molecule target inhibitors and is unlikely to induce resistance because it efficiently inhibits KRAS and its downstream effector signaling pathways.

In *in vivo* experiments, we newly demonstrated that MIR143#12 induced neoangiogenesis in the marginal region of tumors near the injection site. Although this result suggests a positive effect on tumor growth, necrotic areas appeared around the capillaries. It has been reported that MIR143-3p induces angiogenesis by targeting VASH1, the negative regulating factor of angiogenesis in lung cancer.^22^ Therefore, further studies are warranted on the effects of MIR143#12 on angiogenesis in tumors.

Although the effects of MIR143#12 on growth of normal cells were negligible, development of a DDS that delivers MIR143#12 in a tumor-specific manner is important from the viewpoint of side effects. However, in recent years, several nucleic acid drugs, such as patisiran^35^ and the mRNA coronavirus disease 2019 (COVID-19) vaccine,^36^^,^^37^ have been approved for clinical use and use lipo-nano particles as the DDS. With development of these drugs, nucleic acid medicine has been attracting increasing attention. Patisiran, an RNA interference treatment agent, is used to treat transthyretin familial amyloidosis by specifically inhibiting transthyretin synthesis in the liver.^35^ Regarding innovative development of anticancer nucleic acid medicines, further studies are needed on tumor-specific DDSs from the viewpoint of efficacy and side effects.

## Materials and methods

### Cell lines and cell viability

The WiDr, SW480, DLD-1, SW837, PANC-1, MIA PaCa-2, ASF 4-1, TIG-3-20, and KMST-6 cell lines were obtained from the Japanese Cancer Research Resources Bank. The SW48 cell line was from the Cell Resource Center for Biomedical Research of Tohoku University. The HT-29 and H9c2 cell lines were procured from the American Type Culture Collection. The BxPC-3 cell line was obtained from the European Collection of Authenticated Cell Cultures. RPMI-1640 medium (Wako Pure Chemicals Industries) used to culture SW48, WiDr, HT-29, SW480, DLD-1, SW837, and BxPC-3 cells; Eagle’s minimum essential medium (Wako) for MIA PaCa-2, PANC-1, ASF 4-1, and TIG-3-20 cells and Dulbecco’s minimum essential medium (Wako) for H9c2 cells. All media were supplemented with 10% (v/v) heat-inactivated FBS (Nichirei Biosciences, Tokyo, Japan), and cells were incubated under an atmosphere of 95% air and 5% CO_2_ at 37°C. Testing for *Mycoplasma* contamination was performed using the activity of certain mycoplasmal enzymes (MycoAlert *Mycoplasma* detection kit; Lonza, Basel, Switzerland). The number of viable cells was assessed using the trypan blue dye exclusion test.

### Transfection experiments

All cells were seeded on 6-well plates at a concentration of 0.5 × 10^5^ cells per well (10%–30% confluence) the day before transfection. The mature type of MIR143#12 or siRNAs for *KRAS*, *AKT*, and *ERK2* (siR-KRAS and siR-ERK2; Invitrogen, Carlsbad, CA, USA; siR-AKT, Cell Signaling Technology, Danvers, MA, USA) were used for transfection of cells, which was achieved using the cationic lipid product Lipofectamine RNAiMAX reagent (Invitrogen) according to the manufacturer’s Lipofection protocol. The non-specific control RNA (HSS, Hokkaido, Japan) sequence was 5′-GUAGGAGUAGUGAAAGGCC-3′, which was used as a control for non-specific effects. The sequence of MIR143#12 used in the present study has been reported previously.^15^ The sequence of siR-KRAS was 5′-AAUGCAUGACAACACUGGAUGACCG-3′, and that of siR-ERK2 was 5′-CAAGUGCCACAUGCCUACGAUUGAA-3′. The effects of transfection of MIR143#12 and siRNAs into cells were assessed after 48, 72, or 96 h.

### qRT-PCR

Total RNA was isolated from cultured cells using NucleoSpin miRNA (TaKaRa, Otsu, Japan). RNA concentration and purity were assessed by UV spectrophotometry. RNA integrity was checked by formaldehyde gel electrophoresis. Total RNA from normal epithelial tissues from colon and pancreas were human colon total RNA and human pancreas total RNA (TaKaRa). To assess the expression levels of MIR143-3p, we conducted qRT-PCR using TaqMan miRNA assays (Applied Biosystems) and the Thunderbird Probe qPCR Mix (Toyobo, Osaka, Japan) according to the manufacturer’s protocol. RNU6B was used as an internal control. Relative expression levels were calculated using the ΔΔCt method.

### Assay for the stability of miRNA *in vitro* and *in vivo*

Assays for the stability of miRNA *in vitro* were performed as described previously.^38^ We incubated 20 pmol of the mirVana miRNA mimic has-miR-143-3p (Thermo Fisher Scientific), MIR143#1, or MIR143#12 without Lipofectamine RNAiMAX reagent in 100 μL of FBS at 37°C for 0, 5, 30, or 60 min. Total RNA was isolated, and qRT-PCR was performed using total RNA.

### Western blotting

Cell lysates were prepared with 1% SDS buffer. Tissues were homogenized in chilled lysis buffer comprising 10 mM Tris-HCl (pH 7.4), 1% NP-40, 0.1% deoxycholic acid, 0.1% SDS, 150 mM NaCl, 1 mM EDTA, 2% protease inhibitor cocktail, 2% phosphatase inhibitor cocktail II, and 2% phosphatase inhibitor cocktail III (Sigma-Aldrich) and then left to stand on ice for 20 min. After centrifugation at 13,000 rpm (16,200 × *g*) at 4°C for 20 min in both lysates using himac CT15RE (Hitachi, Tokyo, Japan), supernatants were collected as protein samples. Protein content was measured with the DC Protein Assay Kit (Bio-Rad, Hercules, CA, USA). One microgram of the lysate protein was separated by SDS-PAGE using 10.0% or 12.5% polyacrylamide gels and electroblotted onto Immobilon-P transfer membranes of polyvinylidene fluoride (PVDF) membranes (Merck Millipore, Tullagreen, Carrigtwohill, Cork, Ireland). After blockade of non-specific binding sites for 1 h with 20% PVDF blocking reagent for Can Get Signal (Toyobo) in distilled water, the membrane was incubated at 4°C overnight with primary antibodies using Can Get Signal Solution 1 (Toyobo). The next day, the membrane was washed 3 times with Tris-buffered saline (TBS) containing 0.1% Tween 20 (TBS-T), incubated with secondary antibodies at room temperature for 1 h using Can Get Signal Solution 2 (Toyobo), and then washed 3 times with TBS-T. Immunoblots were visualized using Immobilon Forte Western HRP Substrate (Millipore). The following primary antibodies were used: anti-AKT, phospho-AKT (Ser473), ERK1/2, phospho-ERK1/2, glyceraldehyde-3-phosphate dehydrogenase (GAPDH), PARP, LC3B, Cyclin D1, ERK5, SOS1, cleaved PARP, CD31, and p-Histone H3 (Cell Signaling Technology). Anti-KRAS (LifeSpan BioScience, Seattle, WA, USA), anti-β-actin (Sigma-Aldrich), and horseradish-peroxidase (HRP)-conjugated goat anti-rabbit and horse anti-mouse IgG (Cell Signaling Technology) were used as secondary antibodies. GAPDH and β-actin were used as internal controls. Immunoblot images were acquired using the ImageQuant LAS4000 biomolecular imager (GE Healthcare Life Sciences, Pittsburgh, PA, USA).

Densitometry analysis was performed using ImageQuant Total Lab-7 (GE Healthcare Life Sciences) image analysis software. Band densitometry intensities between different samples were normalized against β-actin or GAPDH.

### IC50 calculation

IC50 calculation was performed as described previously.^39^

The concentration was plotted on the x axis, and cell viability was plotted on the y axis. Then, using the value of higher and lower sides of 50% of concentration and cell viability, a linear equation was created as follows:

IC50 = 10ˆ(log(A/B) × (50-C)/(D-C) + log(B)), where

A is the concentration of the higher side of 50% of cell viability, B is the concentration of the lower side of 50% of cell viability, C is cell viability at the concentration of B, D is cell viability at the concentration of A, and ˆ is the symbol of power in the Excel software.

### Ago2 immunoprecipitation

To validate the uptake of MIR143#12 into the RISC, we used the MagCapture microRNA Isolation Kit, Human Ago2 (Fujifilm). Extraction of miRNA bound to Ago2 in the RISC was performed according to the manufacturer’s instructions. qRT-PCR was performed using miRNA extracts.

### Animal experiments

Animal experimental protocols were approved by the Committee for Animal Research and Welfare of Gifu University. BALB/cAJcl-nu/nu (nude) mice (female, 4 weeks old) were obtained from CLEA Japan (Tokyo, Japan). Human colorectal cancer DLD-1 cells were inoculated at 2.0 × 10^6^ cells/100 μL per site into the back of each mouse. The inoculation day was set as day 0. Ten days after inoculation, we confirmed engraftment of tumors. miRNA was mixed with Lipofectamine RNAiMAX reagent for administration to mice. After control RNA or MIR143#12 (160 μg/kg per 1 administration) in 46 μL of Opti-MEM had been incubated with 3 μL of Lipofectamine RNAiMAX reagent, the mixture was injected into tumors every day. Each group contained 8 mice. Injections were stopped when tumors disappeared. Tumor volumes were calculated using the following formula: 0.5236 L_1_ (L_2_)2, where L_1_ is the long axis and L_2_ is the short axis of the tumor.

### Histology and immunohistochemistry

Histology and immunohistochemistry were performed as described previously.^40^ Tissues for H&E staining and immunohistochemistry were fixed in 4% neutral-buffered paraformaldehyde, embedded in paraffin, sectioned at a thickness of 5 μm, and mounted on microscope glass slides (S2226 for H&E and SCRE-05 for immunohistochemistry, Matsunami Glass). Slides were deparaffinized in Lemosol and rehydrated by passages through a graded series of alcohol. H&E staining was performed with Tissue-Tek Hematoxylin 3G (9131-4P, Sakura Finetek Japan) and Tissue-Tek Eosin (8659, Sakura Finetek Japan). Regarding immunohistochemistry, sections were incubated in 0.3% hydrogen peroxide in methanol for 20 min to inhibit endogenous peroxidase activity. Sections were then autoclaved at 120°C for 1 min in antigen retrieval buffers (Immunoactive, IA6500, IA9500, Matsunami Glass) for antigen retrieval and cooled to room temperature. After 3 washes with TBS-T, sections were blocked with 2.5% normal horse serum (S-2012-50, Vector Laboratories) and incubated with primary antibodies at 4°C overnight. Sections were then washed 3 times with TBS-T and incubated for 20 min with the secondary antibody conjugated with HRP (ImmPRESS HRP Horse Anti-Rabbit IgG Polymer Detection Kit, MP-7401, Vector Laboratories). After 3 washes with TBS-T, signals were visualized with 3',3-diaminobenzidine (DAB) chromogen solution (ImmPACT DAB Substrate, SK-4105, Vector Laboratories). Slides were counterstained using hematoxylin or Giemsa staining. Histopathological images were acquired using a microscope (BZ-X700, Keyence).

### *In situ* hybridization

Tissues for *in situ* hybridization were immersed in Tissue-Tek OCT compound (Sakura Finetek Japan) in a cryomold (Sakura Finetek Japan) and frozen with liquid nitrogen. Frozen sections were cut to a thickness of 10 μm using a cryostat and mounted on microscope glass slides. Samples were stored at −80°C. Frozen sections were fixed in 4% neutral-buffered paraformaldehyde. The concentration of the hybridization miR-143-3p probe (YD00610001, QIAGEN) was 40 nM. The hybridization probe (50 μL) was added to each slide. Slides were hybridized at 53°C for 1 h using a hybridizer (Dako Hybridizer, Dako Agilent). The probe was removed, and slides were placed in 0.2% saline sodium citrate (SSC) three times at room temperature for 5 min. All SSC solutions were made fresh from a 20× SSC stock solution (15557044, Thermo Fisher Scientific).

### Quantification and statistical analysis

Each examination was performed in triplicate. Regarding *in vitro* and *in vivo* experiments, the significance of differences was assessed using two-sided Student’s t test. We performed statistical analyses using Pearson’s correlation coefficient and the TDIST function of Excel. Values are shown as the mean ± standard deviation. p < 0.05 was considered significant.

### Data availability statement

Data sharing is not applicable to this article because no datasets were generated or analyzed during the current study.

## Supplemental information

Document S1. Figures S1–S3

Document S2. Article plus supplemental information
