# Supplementary material for: Chemically modified MIR143-3p exhibited anti-cancer effects by impairing the KRAS network in colorectal cancer cells
Source: Mol Ther Nucleic Acids. 2022 Sep 7;30:49–61. doi: 10.1016/j.omtn.2022.09.001 (PMC9507988; doi:10.1016/j.omtn.2022.09.001)
Supplement: Document S1. Figures S1–S3 [file mmc1.pdf]

OMTN, Volume *30*

Supplemental information

Chemically modified MIR143-3p exhibited anti-cancer effects by impairing the KRAS network in colorectal cancer cells

Nobuhiko Sugito, Kazuki Heishima, and Yukihiro Akao

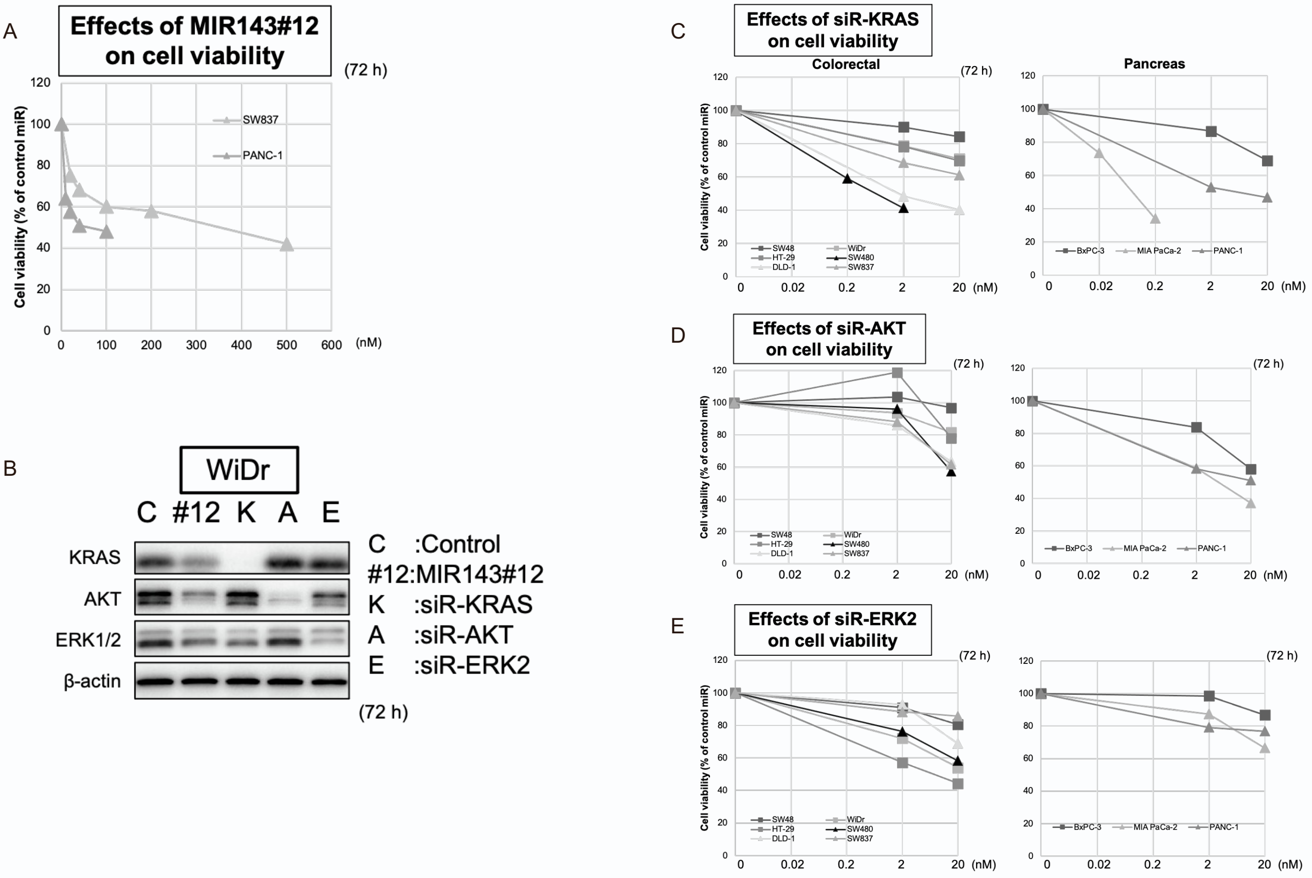

Supplementary Figure S1. **MIR143#12, siR-KRAS, siR-AKT, and siR-ERK2 inhibited the growth of colorectal and pancreatic cancer cells with or without KRAS mutations. Related to the Table and Figure 2.**

(A) Effects of the ectopic expression of MIR143#12 at concentrations higher than 20 nM on the viability of SW837 and PANC-1 cells at 72 hours. (B) The RNA interference of MIR143#12, siR-KRAS, siR-AKT, or siR-ERK2 was evaluated by the expression of each gene in the WiDr cell line. (C-E) Effects of the ectopic expression of siR-KRAS (C), siR-AKT (D), or siR-ERK2 (E) on the viability of colorectal or pancreatic cancer cell lines at 72 hours.

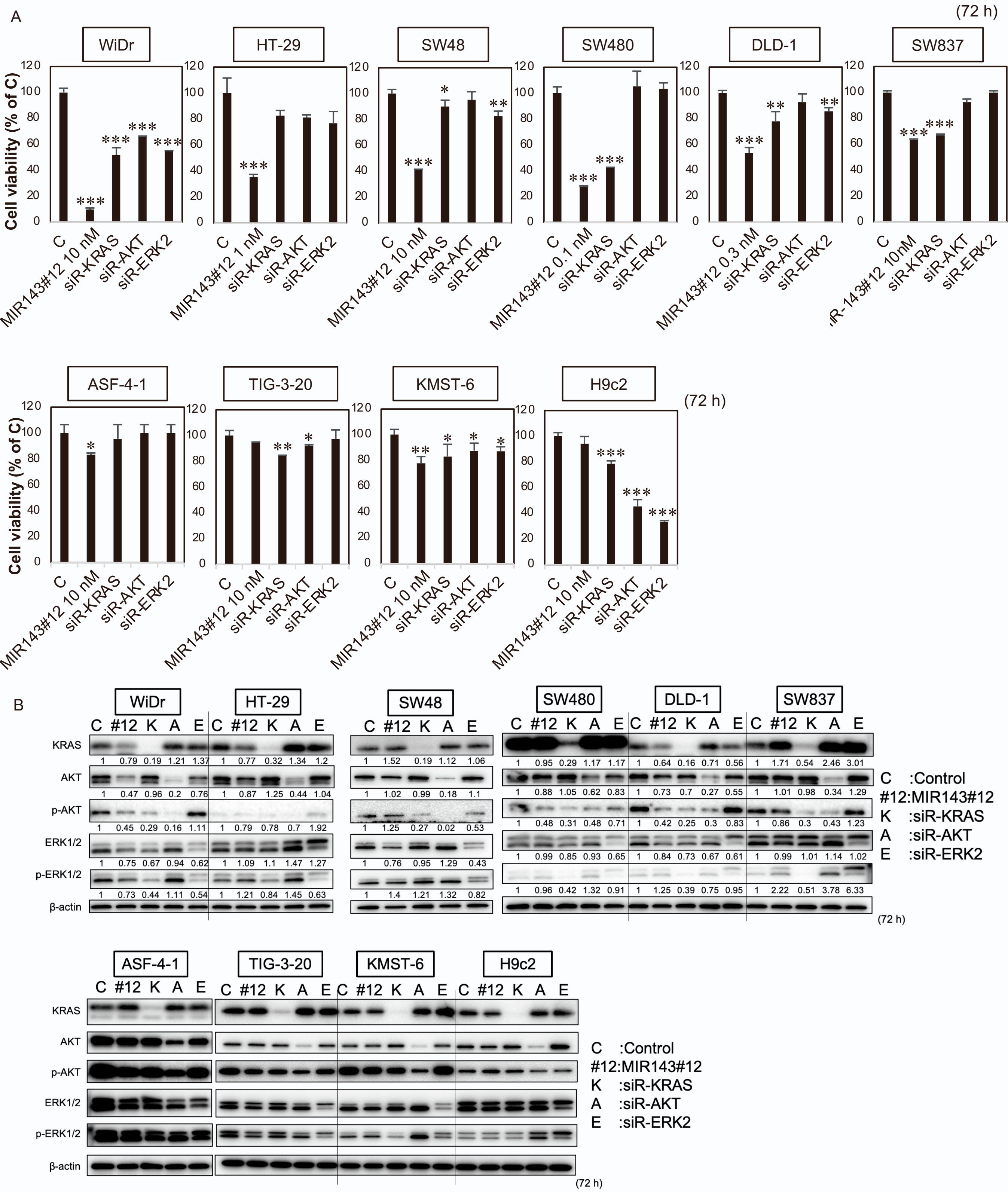

Supplementary Figure S2. **MIR143#12 was more effective than siR-KRAS, siR-AKT, and siR-ERK2 in colorectal cancer cell lines. Related to Figure 3.**

(A, B) We used concentrations of IC50 of MIR143#12 against each cell line (Table 1), and the same concentration was used for ASF-4-1, TIG-3-20, KMST-6, H9c2, and each colorectal cancer cell line. (A) Effects of the ectopic expression of MIR143#12, siR-KRAS, siR-AKT, and siR-ERK2 on the viability of ASF-4-1, TIG-3-20, KMST-6, H9c2, and colorectal cancer cell lines at 72 hours. (B) The effects of RNA interference by each RNA was evaluated by Western blots. Some blot data from WiDr cell lines were used in Figure S1B. Results are shown as the mean ± SD; * p<0.05, ** p<0.01, *** p < 0.001.

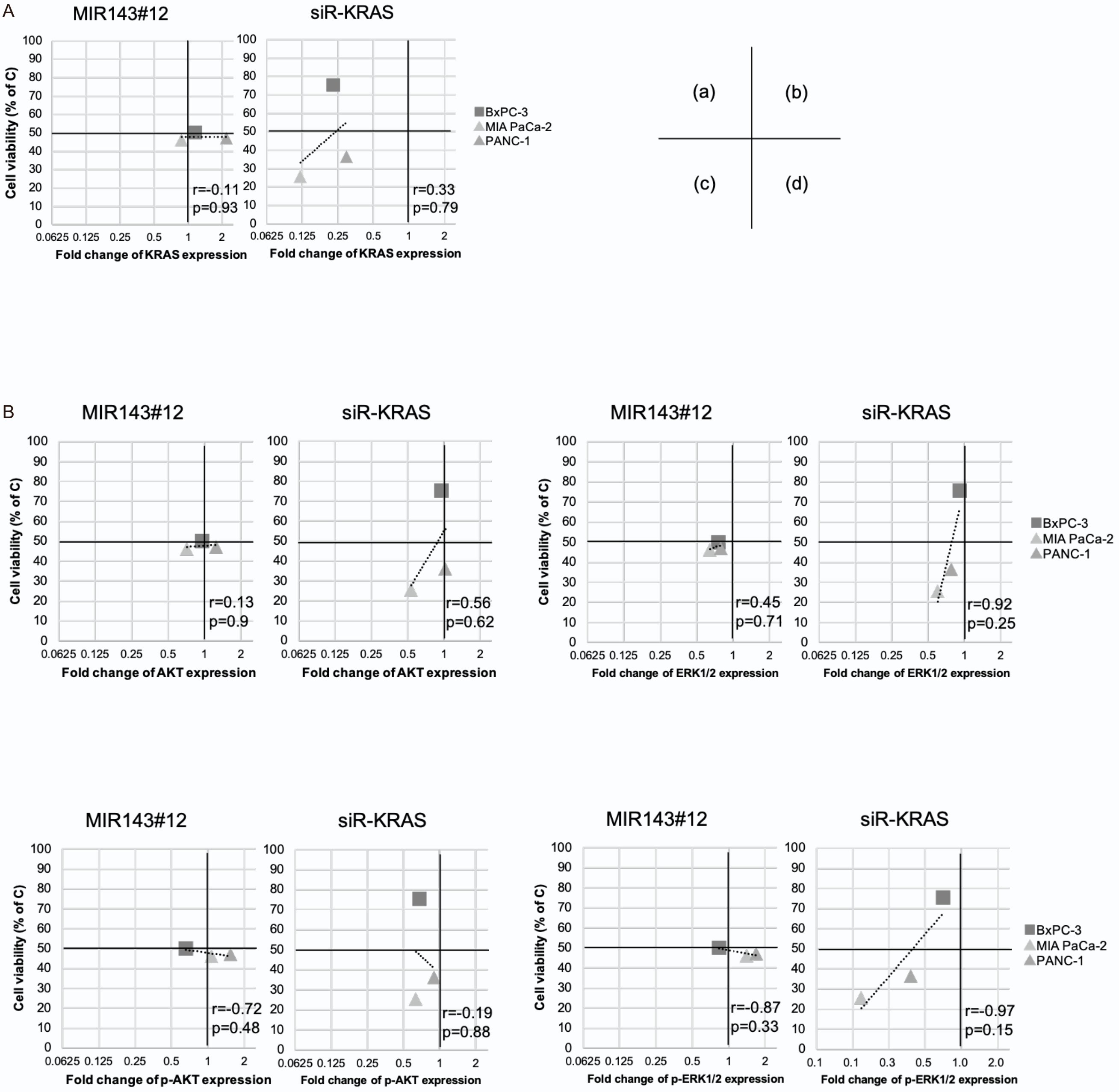

Supplementary Figure S3. **A correlation analysis of pancreatic cancer cell lines. Related to Figure 4.**
(A, B) The relationship between the viable cell rate and expression levels of KRAS (A), AKT, and ERK (B) after transfection with MIR143#12 or siR-KRAS in pancreatic cancer cell lines. We performed a statistical analysis using Pearson's correlation coefficient and the TDIST function of Excel. A p value <0.05 was considered to be significant. Values of r from 0.5 to 0.7 showed a positive correlation, values from 0.7 to 1.0 a strong positive correlation, and values of r from -0.7 to -1.0 a strong negative correlation.
